# Genetic risk factors modulate the association between physical activity and colorectal cancer

**DOI:** 10.21203/rs.3.rs-7350654/v1

**Published:** 2025-09-02

**Authors:** Anita R. Peoples, Mireia Obón-Santacana, Andre E. Kim, Eric S. Kawaguchi, Yubo Fu, Conghui Qu, Ferran Moratalla-Navarro, John Morrison, Yi Lin, Volker Arndt, Sonja I. Berndt, Stephanie A Bien, D. Timothy Bishop, Emmanouil Bouras, Hermann Brenner, Daniel D. Buchanan, Peter T. Campbell, Andrew T. Chan, Jenny Chang-Claude, David V. Conti, Douglas AC. Corley, Matthew A. Devall, Niki Dimou, David A. Drew, Stephen B. Gruber, Marc J. Gunter, Sophia Harlid, Tabitha A. Harrison, Michael Hoffmeister, Li Hsu, Jeroen R. Huyghe, Temitope O. Keku, Anshul Kundaje, Juan Pablo Lewinger, Li Li, Brigid M. Lynch, Loic Le Marchand, Vicente Martín, Neil Murphy, Christina C. Newton, Shuji Ogino, Sheetal Hardikar, Jennifer Ose, Rish K. Pai, Julie R. Palmer, Nikos Papadimitriou, Bens Pardamean, Andrew J. Pellatt, Mila Pinchev, Elizabeth A. Platz, John D. Potter, Gad Rennert, Edward A. Ruiz-Narvaez, Lori C. Sakoda, Robert E. Schoen, Anna Shcherbina, Mariana C. Stern, Yu-Ru Su, Claire E. Thomas, Yu Tian, Konstantinos K. Tsilidis, Caroline Y. Um, Franzel J. B. van Duijnhoven, Bethany van Guelpen, Kala Visvanathan, Jun Wang, Emily White, Alicja Wolk, Michael O. Woods, Anna H. Wu, Cornelia M. Ulrich, Ulrike Peters, W. James Gauderman, Victor Moreno

**Affiliations:** American Cancer Society; Catalan Institute of Oncology (ICO), L’Hospitalet del Llobregat; University of Southern California; University of Southern California; University of Southern California; Fred Hutchinson Cancer Center; Catalan Institute of Oncology (ICO), L’Hospitalet del Llobregat; University of Southern California; Fred Hutchinson Cancer Center; German Cancer Research Center (DKFZ); National Cancer Institute, National Institutes of Health; Fred Hutchinson Cancer Center; University of Leeds; University of Ioannina School of Medicine; German Cancer Research Center (DKFZ); The University of Melbourne; Albert Einstein College of Medicine; Massachusetts General Hospital, Harvard Medical School; German Cancer Research Center (DKFZ); University of Southern California; Kaiser Permanente Northern California; University of Virginia; International Agency for Research on Cancer, World Health Organization; Massachusetts General Hospital, Harvard Medical School; City of Hope National Medical Center; International Agency for Research on Cancer, World Health Organization; Umeå University; Fred Hutchinson Cancer Center; German Cancer Research Center (DKFZ); Fred Hutchinson Cancer Center; Fred Hutchinson Cancer Center; University of North Carolina; Stanford University; University of Southern California; University of Virginia; Cancer Council Victoria; University of Hawaii Cancer Center; Universidad de León; International Agency for Research on Cancer, World Health Organization; American Cancer Society; Broad Institute of MIT and Harvard; Huntsman Cancer Institute; University of Applied Sciences and Arts; Mayo Clinic Arizona; Slone Epidemiology Center at Boston University; International Agency for Research on Cancer, World Health Organization; Binus University; University of Texas MD Anderson Cancer Center; Lady Davis Carmel Medical Center; Johns Hopkins Bloomberg School of Public Health; Fred Hutchinson Cancer Center; Lady Davis Carmel Medical Center; University of Michigan School of Public Health; Kaiser Permanente; University of Pittsburgh Medical Center; Stanford University; University of Southern California; Kaiser Permanente San Francisco Medical Center; Fred Hutchinson Cancer Center; German Cancer Research Center (DKFZ); University of Ioannina School of Medicine; American Cancer Society; Wageningen University & Research; Umeå University; Johns Hopkins Bloomberg School of Public Health; University of Southern California; Fred Hutchinson Cancer Center; Karolinska Institutet; Memorial University of Newfoundland, St. John’s; University of Southern California; Huntsman Cancer Institute; Fred Hutchinson Cancer Center; University of Southern California; Catalan Institute of Oncology (ICO), L’Hospitalet del Llobregat

**Keywords:** physical activity, gene-environment interaction, colorectal cancer, GWAS

## Abstract

**Background:**

Physical activity (PA) is an established protective factor for colorectal cancer (CRC), but it is unclear if genetic variants modify this effect. To investigate this possibility, we conducted a genome-wide gene–PA interaction analysis.

**Methods:**

Using logistic regression and two-step and joint tests, we analyzed interactions between common genetic variants across the genome and PA in relation to CRC risk. Self-reported PA levels were categorized as active (≥ 8.75 MET-h/wk) vs. inactive (< 8.75 MET-h/wk) and as study- and sex-specific quartiles of activity.

**Results:**

PA had an overall protective effect on CRC (OR [active vs. inactive] = 0.85; 95%CI = 0.81–0.90). The two-step GxE method identified an interaction between rs4779584, an intergenic variant near the *GREM1* and *SCG5* genes, and PA for CRC risk (p-interaction = 2.6×10^− 8^). Stratification by genotype at this locus showed a significant reduction in CRC risk by 20% in active vs. inactive participants with the CC genotype (OR = 0.80; 95%CI = 0.75–0.85), but no significant PA–CRC association among CT or TT carriers. When PA was modeled as quartiles, the 1-d.f. GxE test identified that rs56906466, an intergenic variant near the *KCNG1* gene, modified the association between PA and CRC (p-interaction = 3.5×10^− 8^). Stratification at this locus showed that increase in PA (highest vs. lowest quartile) was associated with a lower CRC risk solely among TT carriers (OR = 0.77; 95%CI = 0.72–0.82).

**Conclusions:**

In summary, we identified two genetic variants that modified the association between PA and CRC risk. One of them, related to *GREM1* and *SCG5*, suggests that the bone morphogenetic protein (BMP)-related, inflammatory, and/or insulin signaling pathways may be associated with the protective influence of PA on colorectal carcinogenesis.

## BACKGROUND

Colorectal cancer (CRC) is a major global cause of morbidity and mortality. It is the third most commonly diagnosed cancer and second leading cause of death in the world, with more than 1.9 million incident cases and 0.9 million deaths in 2020 [1]. It is predicted that there will be 2.2 million and 3.2 million new CRC cases by 2030 [2] and 2040 [3], respectively, confirming CRC as a major continuing public health burden. The underlying etiology of CRC is multifactorial with a combination of genetic and environmental factors increasing the likelihood of developing CRC [4]. Among these risk factors, physical activity, a lifestyle factor, is an established protective factor against CRC [5–9].

Multiple observational studies and several systematic reviews have shown that regular physical activity (occupational or leisure time) is a modifiable factor associated with lower CRC risk [10–13]. In particular, the World Cancer Research Fund/American Institute for Cancer Research (WCRF/AICR) Continuous Update Project reported lower CRC risk with increased physical activity and classified the evidence linking physical activity to lower CRC risk as ““strong” [5]. Despite the beneficial health effects of physical activity, a recent study reported that more than a quarter of all adults globally were not getting sufficient physical activity [14].

There is substantial understanding of the mechanisms underlying the protective association of physical activity with CRC risk, for example, physical activity is known to have beneficial effects on skeletal muscle mass, immune function, sleep, and mental health [7, 15–21]. Physical activity also reduces obesity (fat mass), which has a beneficial effect on CRC through a reduction in insulin resistance and inflammation, both of which have been associated with CRC development [7, 22–24]. More recently, physical activity has been linked to improved gut microbiome diversity [25]. Further, non-modifiable genetic factors may play a role between physical activity and CRC. However, only a few gene-environment (GxE) interaction studies to date have investigated the association of physical activity with CRC risk according to genetic variants [26–29], all of which were limited by small sample size or restricted to candidate genes/pathways.

Understanding the genetic factors that may influence the relationship between physical activity and CRC risk can offer novel insights into potential biological mechanisms of colorectal carcinogenesis, as well as better inform efforts to promote physical activity and potentially identify individualized physical activity prescriptions. We conducted the largest genome-wide GxE analysis to date, aiming to identify novel genetic variants that may modify the protective association between self-reported physical activity and CRC risk in order to obtain insight into potential mechanisms behind this association.

## METHODS

### Study participants

The study included individual level genomic and epidemiologic data from three CRC consortia: the multi-centered Colon Cancer Family Registry (CCFR), the Genetics and Epidemiology of Colorectal Cancer Consortium (GECCO), and the Colorectal Cancer Transdisciplinary Study (CORECT), which have been previously described [30–35]. Nested case-control sets were assembled from cohort studies. Control participants were matched on age, sex, and enrollment date/trial group, when applicable. CRC cases were defined as invasive colon or rectal tumors and were confirmed via multiple sources including electronic medical records, pathology reports, state or provincial cancer registries, and/or death certificates. For the small subset of advanced adenomas (7–8%), matched controls were polyp-free and were confirmed by sigmoidoscopy or colonoscopy at the time of adenoma diagnosis. Each study was approved by relevant ethics committees or review boards from respective institutions. All participants provided written informed consent at recruitment.

### Data harmonization

Data were collected and centralized at the GECCO consortium coordinating center at the Fred Hutchinson Cancer Center [34]. Briefly, data harmonization consisted of a multi-step procedure, in which common data elements (CDEs) were defined *a priori* for data harmonization. Study questionnaires and data dictionaries were examined and, through an iterative process of communication with data contributors, elements were mapped to these CDEs. Definitions, permissible values, and standardized coding were implemented into a single database via SAS and T-SQL. Resulting data were checked for errors and outlying values within and between studies [36].

### Epidemiologic and lifestyle data collection

Information on demographic, lifestyle, and environmental factors as well as potential risk factors such as age at diagnosis or enrollment, sex, education level, smoking status, total energy consumption (kcal/day), and self-reported or measured weight and height were collected via in-person interviews or through structured self-administered questionnaires in each study. Total energy consumption was derived from the Food Frequency Questionnaires, with missing values imputed by study-sex-specific means. Body mass index (BMI) was calculated using the weight (kg) and height (m) of each participant.

### Physical activity exposure measure

Information on physical activity was obtained from structured questionnaires, such as the International Physical Activity Questionnaire (IPAQ) short form [37], European Prospective Investigation into Cancer and Nutrition (EPIC) physical activity questionnaire, and Nurses’ Health Study physical activity questionnaire, among others. Physical activity was estimated in metabolic equivalent tasks hours per week (MET-h/wk), which was derived for each participant, to determine the approximate average amount of time per week that the individual spent in leisure activities or all activities if leisure was not specified.

Moderate activity was defined as 3.5 to 6 MET-h/wk and vigorous activities as ≥ 6 MET-h/wk [38]. Thus, at least 8.75 MET-h/wk approximately corresponds to the current physical activity guidelines of a minimum of 150 minutes (= 2.5 hours) of moderate or 75 minutes of vigorous activity per week as recommended for individuals with cancer or for cancer prevention [39–42]. Based on these guidelines and previously published literature in CRC [43–45], the participants in the present study were categorized into two groups: active (≥ 8.75 MET-h/wk) vs. inactive (< 8.75 MET-h/wk; reference category). Because the majority of the participants were active, we also calculated study- and sex-specific quartiles for physical activity as a secondary variable, where the quartile groups were coded as 1, 2, 3, or 4, respectively. This variable was treated as continuous (change in one quartile) when assessing the association between physical activity and CRC, and as categorical (1st quartile as reference group) in the genome-wide scans.

### Genotyping, quality control, and imputation

Detailed information on genotyping, imputation, and quality control have been described previously [30, 32]. In brief, genotyped single nucleotide polymorphisms (SNPs) were excluded based on deviation from Hardy-Weinberg Equilibrium (p < 1×10^− 4^), low call rate (< 95–98%), discrepancies between reported and genotypic sex, and discordant calls between duplicates. Autosomal SNPs in all studies were imputed to the Haplotype Reference Consortium (HRC) r1.1 (2016) panel using the University of Michigan Imputation Server [46] and treated as dosage for data management and analyses using R package BinaryDosage [47]. Imputed common SNPs were excluded if they had low imputation quality (*R*^2^ < 0.8) and pooled minor allele frequency (MAF) ≤ 1%. After quality control, a total of over > 7.2 million SNPs were used for the gene-environment interaction analysis, noticeably with high redundancy due to linkage disequilibrium (LD).

### Sample size

Analyses were limited to individuals of European ancestry, based on self-reported race and clustering of principal components (PCs) with 1000 Genomes EUR superpopulations [48]. Participants were excluded based on cryptic relatedness or duplicates (prioritizing cases and/or individuals genotyped on the better platform), and genotyping/imputation errors. We also excluded studies that did not collect physical activity data. The pooled sample size for the study- and sex-specific quartile physical activity variable was 42,602 participants from 31 studies (71% prospective cohort studies). For the dichotomous active-inactive physical activity variable, with 8.75 MET-h/wk as the cutoff value, the final pooled sample size was 39,992 participants from 27 studies (74% prospective cohort studies) (**Supplementary Table 1**).

### Statistical Analyses

To evaluate the main effects of physical activity on CRC risk, logistic regression models were conducted for each study, with adjustment for age at diagnosis or enrollment, sex, and total energy consumption (when available). Models with genetic variables were further adjusted for the first three PCs of genetic ancestry to account for potential population substructure. The study-specific results were combined using random-effects meta-analysis methods (Hartung-Knapp) to obtain summary odds ratios (ORs) and 95% confidence intervals (CIs) [49]. The heterogeneity p-values were calculated using Cochran’s Q statistics [50], while funnel plots identified studies with outlying ORs for potential exclusion and sensitivity analyses. Additional models were fitted, stratified by study design (case-control vs. cohort), sex, and tumor site (proximal colon, distal colon, rectal). All meta-analyses were performed using the R package Meta [51].

Genome-wide interaction scans of common markers were conducted in the overall study population to maximize power. For the purposes of this study, *E* indicates physical activity, *G* indicates a particular SNP, *D* indicates CRC disease status, and *C* refers to a set of adjustment covariables. We utilized not only the traditional logistic regression test of GxE (1-degree of freedom test; 1-d.f.), but also the more powerful joint 3-d.f. test [52, 53] and two-step EDGE method [54–56]. The R package GxEScanR [57] was used to perform these analyses.

For the 1-d.f. test, we examined multiplicative interactions by fitting a traditional logistic regression model including an interaction term in the form: *logit* (*Pr* (*D* = 1|*G*)) = *β*. _0_ + *β*_*G*_*G* + *β*_*E*_*E* + *β*_*GxE*_*GxE* + *β*_*C*_*C*, where *H*0 : *β*_*GxE*_ = 0 tests potential departures from multiplicative associations of *E* and *G* on *D*.

We also performed a joint test of association, which can improve power to detect disease susceptibility loci in a wider range of circumstances by accounting for GxE interactions, e.g., in circumstances where susceptibility loci affect only individuals with certain environmental exposure profiles [53, 58]. For this we used the 3-d.f. test of the joint null hypothesis *H*0 = *β*_*G*_ = *β*_*GxE*_ = *γ*_*G*_ = 0, where *β*_*G*_ and *β*_*GxE*_ are the main and interaction effects from the logistic model above and *γ*_*G*_ represents the association between **G** and **E** in the combined case-control sample [53, 59].

We further implemented the two-step EDGE method that assesses GxE interaction tests (step 2) based on ranks of an independent filtering or ranking statistic (step 1) [56]. The two-step method can decrease the multiple testing burden and improve power to detect interaction loci [56, 59, 60], provided that steps 1 and 2 are independent. The original approach uses step 1 ranks to prioritize and partition SNPs into exponentially larger bins of fixed sizes and increasingly more stringent step- 2 significance thresholds. However, when analyzing imputed SNPs, highly correlated markers from the same loci fill the top bins, thereby diminishing statistical power. To address this issue, the original weighted hypothesis-testing framework [61] was modified to accommodate bins of varying sizes while appropriately controlling for type I error [55]. In particular, SNPs were partitioned into bins based on step 1 p-value thresholds *in expectation*, which were calculated using the original predetermined bin sizes (initial bin size of 5 and overall alpha = 0.05) with assumed uniform distribution of 1 million independent tests. For step 2 GxE testing, the influx of correlated markers into each bin was accounted for by correcting for the effective number of tests, which was estimated using principal component analysis (PCA) performed on bin-specific genotype correlation matrices [54, 55, 62]. This modification reduces multiple testing burden and improves statistical power, while preserving the overall type I error rate at 5%. For any SNP achieving significance at the overall type I error rate, we computed its corresponding SNP-specific p-value accounting for both steps 1 and 2 of the EDGE procedure, to allow direct comparison to the standard GWAS threshold of 5 × 10^− 8^ [62].

To follow-up statistically significant interactions, we estimated stratified ORs by modeling physical activity in relation to CRC within genotypic groups and the per-allele increase in genotype in relation to CRC stratified by physical activity. We also assessed the extent of genomic inflation by creating quantile-quantile (Q-Q) plots and calculating the genomic inflation factor (lambda). Additionally, we calculated lambda_1000_, which scales the genomic inflation factor to an equivalent study of 1000 cases and 1000 controls, since as lambda scales according to the sample size [63, 64].

To explore variation in GxE effect strengths of association, we also conducted stratified analyses for novel findings by study design, sex, and tumor site. We conducted a sensitivity analysis including the interaction terms GxBMI and E(= physical activity)xBMI in the model, because BMI it is a potential confounder in the physical activity–CRC association [65].

### Functional follow-up

Regional plots for all statistically significant findings were generated using the command- line version (standalone) of LocusZoom v1.3 [66] to examine, in depth, the magnitudes of association, the extent of association signal due to LD, and chromosomal position of findings relative to genes in the given region. Measures of LD were estimated using study population controls. The putative functional role of these SNPs and those in LD (R^2^ > 0.5) at 500 kb flanking regions were examined relative to their potential contribution to regulate gene expression by their: i) direct association with expression of nearby genes (expression quantitative trait loci (eQTLs); and ii) physical location in regions of chromatin accessibility or histone modifications (variant enhancer loci).

Possible eQTL relationships were explored using: i) the Genotype-Tissue Expression (GTEx v8); and ii) the University of Barcelona and University of Virginia genotyping and RNA sequencing project (BarcUVa-Seq) dataset, which includes normal colon tissue samples from 445 healthy individuals [67]. In addition, the BarcUVa-Seq project has data on physical activity in 352 (79%) participants, which we also used to test both specific eQTLs for physical activity status (active vs. inactive; study- and sex-specific quartile variable) and interactions between SNPs and physical activity on gene expression. The BarcUVA-Seq models were adjusted for age (years), sex, sequencing batch (one to four), and tissue location (left, right, transverse, missing). The putative functional role of SNPs and those in LD (r2 > 0.2) and MAF > 0.01 at 500kb flanking regions were investigated relative to their potential contribution to regulate gene expression by their physical location in regions of chromatin accessibility or histone modifications (variant enhancer loci). We annotated only suggestive eQTLs, i.e., those having a nominal p-value < 0.05.

Details of the functional- annotations analyses have been previously published [68, 69]. Briefly, we used an assay for transposase-accessible chromatin with sequencing (ATAC-seq), DNaseI Hypersensitivity (DHS)-seq, H3K27ac histone ChIP-seq, H3K4me1 histone ChIP-seq datasets of primary tissue from healthy colon and primary-tumor primary tissue samples containing active enhancer elements from Scacheri et al. [70], as well as from three CRC cell lines (SW480, HCT116, COLO205). These datasets were processed through ENCODE ATAC-seq/DNASE-seq [71] and histone ChIP-seq pipelines [72] to perform alignment and peak calling.

### GxE analyses for rare variants

To assess the potential contribution of rare SNPs, we also performed a gene-set-based aggregate tests only for rare SNPs using the Mixed effects Score Test for Interactions (MiSTi) approach [73] as a secondary analysis, as the power for rare SNPs testing usually is low. We examined the interactions of physical activity and aggregated rare SNP sets at the gene and enhancer level using MiSTi (MiSTi R package). We used a Fisher’s combination approach under MiSTi (fMiSTi) to discover GxE interactions [73], after adjusting for age, sex, study, and the first three PCs. Because 25,000 gene regions were tested and this was a secondary analysis, interactions with p < 2×10^− 6^ were considered statistically significant, while whereas those with p < 1×10^− 4^ were considered suggestive.

## RESULTS

### Study population characteristics

The total sample size was n = 39,992 (16,383 CRC cases and 23,609 controls), with 76% classified as active (i.e., ≥ 8.75 MET-h/wk). Detailed descriptive characteristics of the study population are presented in Table 1. Compared to controls, CRC cases were more likely to be older, female, ever smokers, have a higher BMI and total energy consumption, and have a lower education level (each p < 0.001). Descriptive characteristics of the study population for the secondary physical activity variable assessed as study- and sex-specific quartiles are provided in **Supplementary Table 2.**

### Physical activity and CRC risk

We observed that being active (≥ 8.75 MET-h/wk) vs. inactive (< 8.75 MET-h/wk) was associated with a 15% risk reduction in CRC in the overall meta-analysis (OR = 0.85; 95% CI = 0.81–0.90; **Supplementary Fig. 1A; Supplementary Table 3**). Sensitivity analyses showed even greater risk reduction for case-control studies (OR = 0.75; 95% CI = 0.66–0.85) compared to cohort-based studies (OR = 0.88; 95% CI = 0.83–0.93). No evidence for heterogeneity was observed across all studies (*P*_het_=0.64; *I*^2^ = 0%;) or among case-control (*P*_het_=0.36; *I*^2^ = 9%) or cohort-based studies (*P*_het_=0.91; *I*^2^ = 0%). Further, analysis stratified by sex showed a risk reduction in both men (OR = 0.83; 95% CI = 0.76–0.90; *P*_het_=0.56; *I*^2^ = 0%) and women (OR = 0.87; 95% CI = 0.81–0.94; *P*_het_=0.86; *I*^2^ = 0%) when comparing active vs. inactive participants. For tumor site, the strongest inverse associations were observed for distal colon (OR = 0.77, 95% CI = 0.71–0.84; *P*_het_=0.64; *I*^2^ = 0%) and proximal colon (OR = 0.84, 95% CI = 0.81–0.90; *P*_het_=0.46; *I*^2^ = 0%), but not for rectal cancer (OR = 0.94, 95% CI = 0.85–1.04; *P*_he*t*_=0.27; *I*^2^ = 15%) comparing active vs. inactive participants. For physical activity measured as study- and sex-specific quartiles (treated as a continuous variable), we observed similar risk reductions for the overall meta-analysis as well as for stratified analysis by sex (**Supplementary Fig. 1B; Supplementary Table 4**). In dose-response (per-quartile) analyses, inverse associations were also observed for rectal cancer (per quartile OR = 0.95; 95% CI = 0.92–0.98; P_het_<0.001; *I*^2^ = 54%) as well as for distal and proximal colon, with some inter-study heterogeneity observed for case-control studies (*P*_het_<0.001; *I*^2^ = 74%). As we found statistically significant associations between physical activity and CRC for the overall population without significant evidence for heterogeneity, we conducted genome-wide GxE testing in the overall study population to maximize power.

### Genome-wide physical activity-interaction scans for CRC risk

The quantile-quantile (Q-Q) plot for the traditional gene-physical activity interactions for CRC risk using 1-d.f. analysis did not show p-value inflation for either primary and or secondary physical activity variables (**Supplementary Fig. 2**).

**Figure 1:**
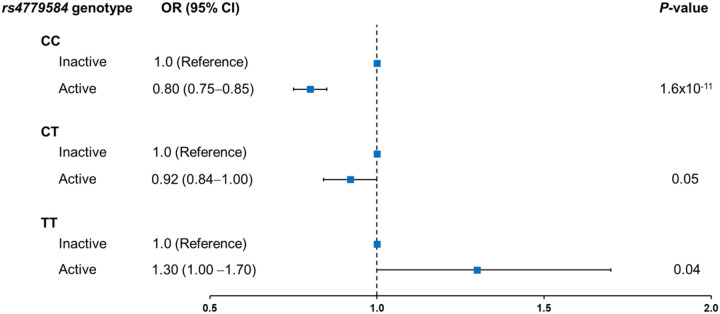
Association between physical activity and colorectal cancer risk stratified by genotype of SNP rs4779584. Physical activity is categorized as active (≥8.75 MET-h/wk) vs. inactive (<8.75 MET-h/wk; reference category).

Table 2 summarizes the statistically significant gene-physical activity interactions identified. Using the two-step EDGE method and the dichotomous physical activity variable (active vs. inactive), we identified statistically significant interactions for 5 SNPs, all of them in LD, on chromosome 15q13.3 located in the intergenic region between Gremlin 1 (*GREM1*) and Secretogranin V (*SCG5*) genes.[74] Among these SNPs with statistically significant interactions, we report only on the interaction of SNP rs4779584 with physical activity in this study (two-step p-value = 2.6×10^− 8^; Table 2), as this SNP was supported by prior evidence on the association with CRC as main effect (per T allele OR: active = 1.20; 95% CI = 1.10–1.20 vs. inactive = 1.00; 95% CI = 0.93–1.10; Table 3).[75] This result was robust in a sensitivity analysis that further accounted for BMI and interactions with BMI, as well as age, sex, study type, total energy consumption, and the first three PCs of genetic ancestry. Specifically, these additional adjustments caused less than a 2% change in the GxPA interaction estimates. Analysis stratified by rs4779584 genotype showed that participants who were physically active vs. inactive had 20% lower CRC risk among those who were carriers of CC (OR = 0.80; 95% CI = 0.75–0.85; p = 1.6x×10^− 11^), while this risk reduction was diminished among those carrying the CT (OR = 0.92; 95% CI = 0.84–1.00) and TT (OR = 1.30; 95% CI = 1.00–1.70;) genotypes (Fig. 1; Table 3). We observed similar interaction effects when analyses were stratified for study type, sex, or tumor site (**Supplementary Table 5**).

The analysis of physical activity assessed as study- and sex-specific quartiles revealed an interaction with one SNP (rs56906466) on chromosome 20q4.5 located near the Potassium Voltage-Gated Channel Modifier Subfamily G Member 1 (*KCNG1*) gene, using the traditional 1-d.f. test (GxE p-value = 3.5×10^− 8^; Table 2; **Supplementary Fig. 3B**). This result was still consistent in a sensitivity analysis that also considered BMI and interactions with BMI along with age, sex, study type, total energy consumption, and the first three PCs of genetic ancestry. As in the previous sensitivity analysis, these adjustments resulted in less than a 2% variation in the GxPA interaction estimates. Analysis stratified by rs56906466 genotype showed statistically significantly lower CRC risk with increases in physical activity, especially when comparing the highest quartile (Q4) to the lowest quartile (Q1), among those who were carriers of TT (OR = 0.77; 95% CI = 0.72–0.82; p = 1.1×10^− 16^). The corresponding inverse associations were not observed for those with TC (Q4 vs. Q1: OR = 1.20; 95% CI = 1.–1.50; p = 0.03) and CC (Q4 vs. Q1: OR = 1.80; 95% CI = 0.65–4.90; p = 0.26) genotypes (Table 3). Similar interactions were observed when analyses were stratified by study type, sex, or tumor site (**Supplementary Table 5**). No other statistically significant interactions were observed (data not shown). Additionally, the GxE analyses for rare variants did not identify any statistically significant interactions. There was also no significant LD-based correlation between rs4779584 and rs56906466 (correlation coefficient, *r*^2^ = 0.001).

### Functional follow-up

Functional annotation analyses around rs4779584 and rs56906466 showed enhanced activities. The SNP rs4779584 and correlated SNPs showed peaks in both normal (i.e., ATAC-seq, H3K4me1) and colon tumor samples (i.e., tumor DHS, tumor H3K27ac) as well as in cancer cell lines (i.e., H3K27ac, H3K4me1). The SNP rs56906466, although not correlated with other SNPs, was identified as a variant enhancer for tumor DHS and cell line DHS (**Supplementary Figs. 4–5**).

Two independent sources of eQTLs analyses were used to expand on the regulatory roles of SNPs rs4779584 and rs56906466. The SNP rs4779584 was observed to be an eQTL in the GTEx v8 compendium as it modified the expression of *GREM1* in liver and pancreas, *SCG5* in liver, and RP11- 758N13.1 in brain, cultured fibroblast, liver, and pancreas tissues. We did not observe any statistically significant eQTL findings for SNP rs56906466.

In relation to the BarcUVa-Seq dataset, which provides colon-specific eQTLs, the SNP in the 15q13.3 region did not modify the expression of *FNM1, GREM1, SCG5*, or other genes in the region (**Supplementary Fig. 4**). Likewise, the models tested in this dataset on the interaction with physical activity measured in the subjects did not reach statistical significance. The same approach was used to assess whether the SNP rs56906466 and the interaction term had eQTL effects on gene expression, but no statistically significant results were observed.

## DISCUSSION

To our knowledge, this is the largest genome-wide study conducted to date to investigate the interactions between variants across the genome and self-reported, harmonized physical activity data. Consistent with previous studies and the WCRF, we observed a statistically significant 15% risk reduction in CRC due to physical activity, similar in magnitude to that previously observed [5, 10–13]. Our analyses identified two novel, statistically significant GxE interactions for physical activity – SNPs rs4779584 and rs56906466 significantly modified the association between physical activity and CRC risk.

The SNP rs4779584, located in the 15q33.3 region, lies between the *GREM1* and *SCG5* genes and has been previously found to contribute to CRC susceptibility [31, 74, 76–79]. Carrying the T allele in rs4779584 has been reported to be associated with an increased CRC risk of 1.26 (95% CI = 1.19–1.34) as compared to the C allele [80]. In our study, we found that physical activity was significantly associated with a lower risk of CRC only among those with the C allele. *GREM1* encodes gremlin 1, which is a signaling protein involved in several pathways relevant to CRC, including the transforming growth factor-β (TGF-β) pathway which has been implicated in tumor invasion and metastasis [81]. *GREM1* is also a proangiogenic factor, suggesting a possible role in cancer development when upregulated [82]. Additionally, Gremlin 1 is an insulin antagonist with elevated levels in type 2 diabetes [83], and has been linked to bone morphogenetic proteins (BMPs) signaling imbalance, which accelerates tumor cell proliferation [84], and is associated with inflammatory processes independently of BMPs [85, 86], *SCG5* encodes secretogranin V (also named 7B2 protein or SGNE1), an essential neuroendocrine signaling molecule that plays a role in cellular proliferation [87, 88]. Although *SCG5* is associated with polyposis syndromes which is linked with CRC risk [89], its direct role in CRC is not as well characterized as compared to *GREM1*’s role in CRC [90]. Further, some studies have also reported a role of *SCG5* in BMI modulation [91, 92]. The identified interactions suggest that the CRC risk reduction due to physical activity may be related to one or several more of these above-mentioned pathways.

There are only a small number of GWAS studies that have identified genetic loci associated with physical activity [93, 94], with one preclinical study suggesting that exercise training epigenetically reprograms *GREM1* expression [95]. However, to our knowledge, no prior studies have reported an interaction between rs4779584 and physical activity on CRC risk. The epidemiologic evidence indicating the beneficial effect of physical activity on CRC risk is extensive, and several biological mechanisms have been identified or proposed, including in some intervention studies, such as physical activity’s effect on immune system, systemic inflammatory markers, energy regulation, hormones levels, insulin resistance, and gut microbial composition [7, 96–98]. Related to our findings, a randomized trial conducted in obese patients who followed different resistance training protocols observed significant reductions in plasma gremlin 1 and C-reactive protein levels compared to a control group [99]. Additionally, myokines (i.e., cytokines), such as myostatin (member of the TGF-β family) or interleukin-6, are secreted by the skeletal muscle in response to intensity training [100, 101]. The effect of regular exercise on *SCG5*, the other gene close to the SNP rs4779584 that showed interactions with physical activity on CRC risk, has been investigated in experimental studies using animal models. However, the results were inconclusive, with one study reported non-significantly decreased SCG5 expression, while the other study reported significantly increased expression levels [102, 103]. Future studies are warranted to describe the plausible biological mechanism by which SNP rs4779584 interacts with physical activity and modifies CRC risk, but on the basis of our findings, genetic markers in this region showed enhanced activity in both normal and tumor samples suggesting a potential regulatory role on transcription of adjacent genes. Consistent with this, we observed that SNP rs4779584 modified the expression of *GREM1* and *SCG5* in pancreas and liver, but not in colon tissue.

We also discovered a new locus rs56906466 located near *KCNG1* that has not been previously associated with CRC, physical activity, or its interaction with physical activity on CRC risk. This gene encodes a member of the large gene family that instructs the building of potassium channels and is abundantly expressed in skeletal muscle. *KCNG1* has been related to insulin secretion, muscle contraction, and neurotransmitter release regulation, among others [104]; however, its functions are not fully understood. Our findings showed that rs56906466 had statistically significant interactions with physical activity in modifying CRC risk. Furthermore, functional-annotations analyses demonstrated that some of the genetic variants interacting with physical activity were located in enhancers and were linked to differential gene expression. However, additional targeted studies will be necessary to further investigate the joint effects of these genes with physical activity on CRC risk.

There is increasing evidence that gene-physical activity interactions (including being physically active or inactive) have an effect on several health-related outcomes such as blood pressure, hypertension, BMI, and insulin metabolism [105]. However, few studies have evaluated the gene-physical activity interaction on CRC risk, and all previous studies followed a candidate-gene approach and included only a limited number of SNPs [26–29]. Two studies evaluated the mediating effects of physical activity on CRC risk via alterations in polymorphisms in the insulin-like growth factor-1 (*IGF-1*) gene, since physical activity is known to modulate IGF-1 serum levels, and observed statistically significant interactions [26, 106]. Khoury-Shakour *et al*. focused their analysis on the polymorphism rs2665802 at intron 4 of the growth hormone 1 (*GH1*) gene and observed that the minor allele A was associated with lower risk of CRC among inactive participants [26]. A recent study assessed the interaction between physical activity and CRC risk based on a polymorphism (rs647161) in the paired-like homeodomain 1 (*PITX1*) gene in a Korean population, and reported a higher risk of CRC among participants who exercised less and carried the minor allele [27]. *PITX1* is considered a tumor suppressor gene [107], and is known to influence the expression of *GH1*, and is related to *IGF-1* [108]. Song *et al*. assessed interactions between physical activity and 31 SNPs (including rs4779584) on CRC risk among 703 CRC cases and 1,406 healthy controls [28]. However, they observed statistically significant interactions only with rs4444235––with increased CRC risk among C carriers who exercised regularly––but not for rs4779584, which may be due to the small sample size. However, none of the above findings could be replicated in the present study (data not shown). Additionally, we observed no LD-based correlation between rs4779584 and rs4444235 (*r*^2^ = 0.004). Given the smaller sample size and candidate gene approach in the study by Song *et al*., it is possible that these are chance findings.

A main strength of our study was a large, well-characterized study population, the largest ever to have examined gene-physical activity interactions. The use of several complementary statistical approaches was also a strength of this study as it allowed detection of specific loci within *GREM1* and *SCG5* and near *KCNG1* genes. However, our findings may not be generalizable outside of European-descent populations as the participants in this study were limited to those with European descent and were far more active than the US general population. The consortium is actively striving to overcome this limitation by expanding our research to encompass other racial and ethnic groups, as well as by harmonizing epidemiological data, which will enable us to expand our future GxE analyses. Additionally, this study included self-report measures of physical activity which are prone to recall and response biases, but these are likely to attenuate ‘true’ associations with disease risk [109]. Lastly, our sample size did not allow us to identify genes, whose rare variants may interact with physical activity and contribute to CRC risk in the aggregate test. Additional functional studies are needed to verify the role of the identified SNPs interacting with physical activity for CRC risk.

## CONCLUSIONS

In conclusion, we identified two novel genetic loci that interact with physical activity to influence CRC risk. Potential mechanisms behind the interaction of rs4779584 and physical activity in CRC risk may be linked in part to the BMP-related, inflammation pathways, and/or insulin signaling in response to physical activity. However, SNP rs56906466 that is near a potassium channel gene, has not been previously described in relation to physical activity or CRC, and additional investigations are required to elucidate the potential mechanisms through which it may be involved in colorectal carcinogenesis, especially in individuals who are not physically active.

## Supplementary Files

This is a list of supplementary files associated with this preprint. Click to download.

- SupplementaryTable1GxEPAv6FINAL.xlsx
- SUPPLEMENTARYTABLESANDFIGURES.docx

## Figures and Tables

**Table 1:** Descriptive characteristics of all study participants by colorectal cancer case-control status with available physical activity data.

Characteristics	Cases (N = 16,383)	Controls (N = 23,609)	*P*-value
**Age (median imputed)** ^a^			
Mean (SD)	65.0 (±9.4)	63.4 (± 8.3)	<0.001
**Sex**			
Female	8,677 (53%)	12,005 (51%)	<0.001
Male	7,706 (47%)	11,604 (49%)	
**Total energy consumption (kcal/day; mean imputed)** ^b,c^			
Mean (SD)	1,967 (±713)	1,910 (±680)	<0.001
**BMI (kg/m^2^)** ^c^			
Mean (SD)	27.2 (± 4.7)	26.9 (± 4.5)	<0.001
**Family history of colorectal cancer** ^c^			
No	10,430 (64%)	12,945 (55%)	0.06
Yes	2,295 (14%)	2,685 (11%)	
**Education level (highest completed)** ^c^			
Less than High School	3,070 (19%)	3,488 (15%)	<0.001
High School/GED	3,366 (21%)	3,161 (13%)	
Some College	3,476 (21%)	5,783 (24%)	
College/Graduate School	5,601 (34%)	8,488 (36%)	
**Ever smoker** ^c^			
No	7,050 (43%)	11,479 (49%)	<0.001
Yes	9,086 (55%)	11,862 (50%)	

NOTE: Data might not add to 100% because of rounding.

Abbreviations: SD, standard deviation; BMI, Body-Mass-Index; GED, General Educational Development Test.

Physical activity categorized as active (≥ 8.75 MET-h/wk) vs. inactive (< 8.75 MET-h/wk; reference category) dichotomous variable.

a Age was assessed at diagnosis or enrollment.

b Calculations exclude individuals with missing total energy intake information.

c Missing values not shown.

*P*-values < 0.05 are statistically significant.

**Table 2:** Results of genome-wide interaction analyses with physical activity for colorectal cancer risk.

Physical Activity Variable	SNP	Chr	BP Position	Locus	Closest Gene	Reference Allele	Alternate Allele	Alternate Allele frequency	Type	Statistical Method	*P*-value GxE^b^
Active / inactive^a^	rs4779584	15	32994756	15q13.3	*GREM1* and *SCG5*	C	T	0.20	intergenic variant	Two-step EDGE	**2.6×10^−8^**
Quartiles^c^	rs56906466	20	49693755	20q4.5	*KCNG1*	T	C	0.06	intron	1-d.f. test	**3.5×10^−8^**

Abbreviations: SNP, single nucleotide polymorphism; Chr, chromosome; BP Position, base pair position based on NCBI Build 37; 1-d.f., 1-degree of freedom.

a Physical activity categorized as active (≥ 8.75 MET-h/wk) vs. inactive (< 8.75 MET-h/wk; reference category).

b *P*-value corresponds to the interaction between genetic variants (G) and physical activity (E) on risk of colorectal cancer in the combined case-control population based on the indicated statistical method.

c Physical activity assessed as study- and sex-specific quartiles.

*P*-values that are statistically significant are indicated in bold text.

Notes: Directly genotyped SNPs were coded as 0, 1, or 2 copies of the count allele. Imputed SNPs were coded as expected gene dosage. Multiplicative interaction terms were modelled as the product of PA and each SNP of interest.

**Table 3:** Associations between physical activity for colorectal cancer risk stratified by genotypes of SNPs of interest.

SNP	Physical Activity	Homozygous non-carriers			Heterozygous			Homozygous carries of the alternate/minor allele			Per alternative allele within strata of Physical Activity cetegories	
		N (Ca/Co)	OR (95% Cl)	*P*-value	N (Ca/Co)	OR (95% Cl)	*P*-value	N (Ca/Co)	OR (95% Cl)	*P*-value	OR (95% Cl)	*P*-value
		CC			CT			TT				
**rs4779584**	Inactive^a^	2,537/3,642	1.00 (Ref.)	-	1,304/1,806	1.00 (0.95–1.10)	0.40	137/228	0.87 (0.69–1.10)	0.23	1.00 (0.93–1.10)	0.98
	Active^a^	7,701/11,960	0.80 (0.75–0.85)	1.6×10^−11^	4,155/5,372	0.95 (0.89–1.00)	0.19	549/601	1.10 (0.99–1.30)	0.08	1.20 (1.10–1.20)	2.0×10^−15^
	Active vs. inactive ^(^by genotype)		0.80 (0.75–0.85)	1.6×10^−11^	-	0.92 (0.84–1.00)	0.05	-	1.30 (1.00–1.70)	0.04		
		TT			TC			CC				
**rs56906466**	Q1^b^	4,168/5,290	1.00 (Ref.)	-	443/715	0.77 (0.67–0.87)	8.0×10^−5^	20/19	1.10 (0.56–2.10)	0.81	0.77 (0.68–0.88)	1.4×10^−4^
	Q2^b^	4,085/5,745	0.91 (0.85–0.96)	0.002	481/710	0.87 (0.76–0.99)	0.03	13/25	0.61 (0.3–1.20)	0.16	0.93 (0.82–1.10)	0.28
	Q3^b^	3,792/5,896	0.81 (0.76–0.86)	6.8×10^−12^	469/669	0.87 (0.77–1.00)	0.047	21/26	1.00 (0.56–1.90)	0.96	1.10 (0.99–1.30)	0.08
	Q4^b^	3,342/5,564	0.77 (0.72–0.82)	1.1×10^−16^	442/637	0.94 (0.82–1.10)	0.35	17/13	1.90 (0.91–4.20)	0.09	1.30 (1.10–1.50)	5.7×10^−4^
	Q2 vs. Q1 (by genotype)^a^	-	0.91 (0.85–0.96)	0.002	-	1.10 (0.95–1.30)	0.16	-	0.56 (0.21–1.50)	0.23		
	Q3 vs. Q1 (by genotype)^a^		0.81 (0.76–0.86)	6.8×10^−12^		1.10 (0.96–1.40)	0.14		0.93 (0.38–2.30)	0.88		
	Q4 vs. Q1 (by genotype)^a^		0.77 (0.72–0.82)	1.1×10^−16^		1.20 (1.00–1.50)	0.03		1.80 (0.65–4.90)	0.26		

Abbreviations: SNP, single nucleotide polymorphism; PA, physical activity; N, number; Ca/Co, case/control; OR, odds ratio; 95% CI, 95% confidence interval. Case/control counts were calculated by imputed genotype probabilities.

a Physical activity categorized as active (≥ 8.75 MET-h/wk) vs. inactive (< 8.75 MET-h/wk; reference category)

b Physical activity, assessed as study- and sex-specific quartiles.

*P*-values that are statistically significant are indicated in bold text.

## Data Availability

The dataset used in the current study may be available from the corresponding author on reasonable request for researchers who meet the criteria for access to confidential data.

## References

[1] Sung H, Ferlay J, Siegel RL, Laversanne M, Soerjomataram I, Jemal A, Bray F: Global Cancer Statistics 2020: GLOBOCAN Estimates of Incidence and Mortality Worldwide for 36 Cancers in 185 Countries. CA Cancer J Clin 2021, 71(3):209–249. 33538338 10.3322/caac.21660

[2] Arnold M, Sierra MS, Laversanne M, Soerjomataram I, Jemal A, Bray F: Global patterns and trends in colorectal cancer incidence and mortality. Gut 2017, 66(4):683–691. 26818619 10.1136/gutjnl-2015-310912

[3] Xi Y, Xu P: Global colorectal cancer burden in 2020 and projections to 2040. Transl Oncol 2021, 14(10):101174. 34243011 10.1016/j.tranon.2021.101174 PMC8273208

[4] Sawicki T, Ruszkowska M, Danielewicz A, Niedzwiedzka E, Arlukowicz T, Przybylowicz KE: A Review of Colorectal Cancer in Terms of Epidemiology, Risk Factors, Development, Symptoms and Diagnosis. Cancers (Basel) 2021, 13(9). 10.3390/cancers13092025 PMC8122718 33922197

[5] World Cancer Research Fund/American Institute for Cancer Research. Continous Update Project Expert Report 2018. Diet, nutrition, physical activity and colorectal cancer. Available at dietandcancerreport.org. In.

[6] Van Blarigan EL, Meyerhardt JA: Role of physical activity and diet after colorectal cancer diagnosis. J Clin Oncol 2015, 33(16):1825–1834. 25918293 10.1200/JCO.2014.59.7799 PMC4438267

[7] Ulrich CM, Himbert C, Holowatyj AN, Hursting SD: Energy balance and gastrointestinal cancer: risk, interventions, outcomes and mechanisms. Nat Rev Gastroenterol Hepatol 2018, 15(11):683–698. 30158569 10.1038/s41575-018-0053-2 PMC6500387

[8] Lauby-Secretan B, Scoccianti C, Loomis D, Grosse Y, Bianchini F, Straif K: Body Fatness and Cancer--Viewpoint of the IARC Working Group. N Engl J Med 2016, 375(8):794–798. 27557308 10.1056/NEJMsr1606602 PMC6754861

[9] Papadimitriou N, Dimou N, Tsilidis KK, Banbury B, Martin RM, Lewis SJ, Kazmi N, Robinson TM, Albanes D, Aleksandrova K : Physical activity and risks of breast and colorectal cancer: a Mendelian randomisation analysis. Nat Commun 2020, 11(1):597. 32001714 10.1038/s41467-020-14389-8 PMC6992637

[10] Kyu HH, Bachman VF, Alexander LT, Mumford JE, Afshin A, Estep K, Veerman JL, Delwiche K, Iannarone ML, Moyer ML : Physical activity and risk of breast cancer, colon cancer, diabetes, ischemic heart disease, and ischemic stroke events: systematic review and dose-response meta-analysis for the Global Burden of Disease Study 2013. BMJ 2016, 354:i3857. 27510511 10.1136/bmj.i3857 PMC4979358

[11] Matthews CE, Moore SC, Arem H, Cook MB, Trabert B, Hakansson N, Larsson SC, Wolk A, Gapstur SM, Lynch BM : Amount and Intensity of Leisure-Time Physical Activity and Lower Cancer Risk. J Clin Oncol 2020, 38(7):686–697. 31877085 10.1200/JCO.19.02407 PMC7048166

[12] McTiernan A, Friedenreich CM, Katzmarzyk PT, Powell KE, Macko R, Buchner D, Pescatello LS, Bloodgood B, Tennant B, Vaux-Bjerke A : Physical Activity in Cancer Prevention and Survival: A Systematic Review. Med Sci Sports Exerc 2019, 51(6):1252–1261. 31095082 10.1249/MSS.0000000000001937 PMC6527123

[13] Morris JS, Bradbury KE, Cross AJ, Gunter MJ, Murphy N: Physical activity, sedentary behaviour and colorectal cancer risk in the UK Biobank. Br J Cancer 2018, 118(6):920–929. 29520109 10.1038/bjc.2017.496 PMC5886126

[14] Guthold R, Stevens GA, Riley LM, Bull FC: Worldwide trends in insufficient physical activity from 2001 to 2016: a pooled analysis of 358 population-based surveys with 1.9 million participants. Lancet Glob Health 2018, 6(10):e1077–e1086. 30193830 10.1016/S2214-109X(18)30357-7

[15] Morley JE, Baumgartner RN, Roubenoff R, Mayer J, Nair KS: Sarcopenia. J Lab Clin Med 2001, 137(4):231–243. 11283518 10.1067/mlc.2001.113504

[16] Kruijsen-Jaarsma M, Revesz D, Bierings MB, Buffart LM, Takken T: Effects of exercise on immune function in patients with cancer: a systematic review. Exerc Immunol Rev 2013, 19:120–143. 23977724

[17] Sitlinger A, Brander DM, Bartlett DB: Impact of exercise on the immune system and outcomes in hematologic malignancies. Blood Adv 2020, 4(8):1801–1811. 32343800 10.1182/bloodadvances.2019001317 PMC7189285

[18] Craft LL, Vaniterson EH, Helenowski IB, Rademaker AW, Courneya KS: Exercise effects on depressive symptoms in cancer survivors: a systematic review and meta-analysis. Cancer epidemiology, biomarkers & prevention : a publication of the American Association for Cancer Research, cosponsored by the American Society of Preventive Oncology 2012, 21(1):3–19. 10.1158/1055-9965.EPI-11-0634 PMC3253916 22068286

[19] Brown JC, Huedo-Medina TB, Pescatello LS, Ryan SM, Pescatello SM, Moker E, LaCroix JM, Ferrer RA, Johnson BT: The efficacy of exercise in reducing depressive symptoms among cancer survivors: a meta-analysis. PLoS One 2012, 7(1):e30955. 22303474 10.1371/journal.pone.0030955 PMC3267760

[20] Kredlow MA, Capozzoli MC, Hearon BA, Calkins AW, Otto MW: The effects of physical activity on sleep: a meta-analytic review. J Behav Med 2015, 38(3):427–449. 25596964 10.1007/s10865-015-9617-6

[21] Takemura N, Cheung DST, Smith R, Deng W, Ho KY, Lin J, Kwok JYY, Lam TC, Lin CC: Effectiveness of aerobic exercise and mind-body exercise in cancer patients with poor sleep quality: A systematic review and meta-analysis of randomized controlled trials. Sleep Med Rev 2020, 53:101334. 32505970 10.1016/j.smrv.2020.101334

[22] Murphy N, Cross AJ, Abubakar M, Jenab M, Aleksandrova K, Boutron-Ruault MC, Dossus L, Racine A, Kuhn T, Katzke VA : A Nested Case-Control Study of Metabolically Defined Body Size Phenotypes and Risk of Colorectal Cancer in the European Prospective Investigation into Cancer and Nutrition (EPIC). PLoS Med 2016, 13(4):e1001988. 27046222 10.1371/journal.pmed.1001988 PMC4821615

[23] Ho GY, Wang T, Gunter MJ, Strickler HD, Cushman M, Kaplan RC, Wassertheil-Smoller S, Xue X, Rajpathak SN, Chlebowski RT : Adipokines linking obesity with colorectal cancer risk in postmenopausal women. Cancer Res 2012, 72(12):3029–3037. 22511581 10.1158/0008-5472.CAN-11-2771 PMC3790260

[24] Zhou B, Shu B, Yang J, Liu J, Xi T, Xing Y: C-reactive protein, interleukin-6 and the risk of colorectal cancer: a meta-analysis. Cancer Causes Control 2014, 25(10):1397–1405. 25053407 10.1007/s10552-014-0445-8

[25] Himbert C, Stephens WZ, Gigic B, Hardikar S, Holowatyj AN, Lin T, Ose J, Swanson E, Ashworth A, Warby CA : Differences in the gut microbiome by physical activity and BMI among colorectal cancer patients. Am J Cancer Res 2022, 12(10):4789–4801. 36381318 PMC9641409

[26] Khoury-Shakour S, Gruber SB, Lejbkowicz F, Rennert HS, Raskin L, Pinchev M, Rennert G: Recreational physical activity modifies the association between a common GH1 polymorphism and colorectal cancer risk. Cancer epidemiology, biomarkers & prevention : a publication of the American Association for Cancer Research, cosponsored by the American Society of Preventive Oncology 2008, 17(12):3314–3318. 10.1158/1055-9965.EPI-08-0062 PMC2665206 19064544

[27] Gunathilake MN, Lee J, Cho YA, Oh JH, Chang HJ, Sohn DK, Shin A, Kim J: Interaction between physical activity, PITX1 rs647161 genetic polymorphism and colorectal cancer risk in a Korean population: a case-control study. Oncotarget 2018, 9(7):7590–7603. 29484135 10.18632/oncotarget.24136 PMC5800927

[28] Song N, Lee J, Cho S, Kim J, Oh JH, Shin A: Evaluation of gene-environment interactions for colorectal cancer susceptibility loci using case-only and case-control designs. BMC Cancer 2019, 19(1):1231. 31849324 10.1186/s12885-019-6456-9 PMC6918639

[29] Morimoto LM, Newcomb PA, White E, Bigler J, Potter JD: Insulin-like growth factor polymorphisms and colorectal cancer risk. Cancer epidemiology, biomarkers & prevention : a publication of the American Association for Cancer Research, cosponsored by the American Society of Preventive Oncology 2005, 14(5):1204–1211. 10.1158/1055-9965.EPI-04-0695 15894673

[30] Huyghe JR, Bien SA, Harrison TA, Kang HM, Chen S, Schmit SL, Conti DV, Qu C, Jeon J, Edlund CK : Discovery of common and rare genetic risk variants for colorectal cancer. Nat Genet 2019, 51(1):76–87. 30510241 10.1038/s41588-018-0286-6 PMC6358437

[31] Peters U, Jiao S, Schumacher FR, Hutter CM, Aragaki AK, Baron JA, Berndt SI, Bezieau S, Brenner H, Butterbach K : Identification of Genetic Susceptibility Loci for Colorectal Tumors in a Genome-Wide Meta-analysis. Gastroenterology 2013, 144(4):799–807 e724. 23266556 10.1053/j.gastro.2012.12.020 PMC3636812

[32] Schmit SL, Edlund CK, Schumacher FR, Gong J, Harrison TA, Huyghe JR, Qu C, Melas M, Van Den Berg DJ, Wang H : Novel Common Genetic Susceptibility Loci for Colorectal Cancer. J Natl Cancer Inst 2019, 111(2):146–157. 29917119 10.1093/jnci/djy099 PMC6555904

[33] Schumacher FR, Schmit SL, Jiao S, Edlund CK, Wang H, Zhang B, Hsu L, Huang SC, Fischer CP, Harju JF : Genome-wide association study of colorectal cancer identifies six new susceptibility loci. Nat Commun 2015, 6:7138. 26151821 10.1038/ncomms8138 PMC4967357

[34] Hutter CM, Chang-Claude J, Slattery ML, Pflugeisen BM, Lin Y, Duggan D, Nan H, Lemire M, Rangrej J, Figueiredo JC : Characterization of gene-environment interactions for colorectal cancer susceptibility loci. Cancer Res 2012, 72(8):2036–2044. 22367214 10.1158/0008-5472.CAN-11-4067 PMC3374720

[35] Peters U, Jiao S, Schumacher FR, Hutter CM, Aragaki AK, Baron JA, Berndt SI, Bezieau S, Brenner H, Butterbach K : Identification of Genetic Susceptibility Loci for Colorectal Tumors in a Genome-Wide Meta-analysis. Gastroenterology 2013, 144(4):799–807.e724. 23266556 10.1053/j.gastro.2012.12.020 PMC3636812

[36] Rolland B, Reid S, Stelling D, Warnick G, Thornquist M, Feng Z, Potter JD: Toward Rigorous Data Harmonization in Cancer Epidemiology Research: One Approach. Am J Epidemiol 2015, 182(12):1033–1038. 26589709 10.1093/aje/kwv133 PMC4675662

[37] Littman AJ, White E, Kristal AR, Patterson RE, Satia-Abouta J, Potter JD: Assessment of a one-page questionnaire on long-term recreational physical activity. Epidemiology 2004, 15(1):105–113. 14712154 10.1097/01.ede.0000091604.32542.97

[38] Nelson ME, Rejeski WJ, Blair SN, Duncan PW, Judge JO, King AC, Macera CA, Castaneda-Sceppa C: Physical activity and public health in older adults: recommendation from the American College of Sports Medicine and the American Heart Association. Med Sci Sports Exerc 2007, 39(8):1435–1445. 17762378 10.1249/mss.0b013e3180616aa2

[39] Piercy KL, Troiano RP, Ballard RM, Carlson SA, Fulton JE, Galuska DA, George SM, Olson RD: The Physical Activity Guidelines for Americans. Jama 2018, 320(19):2020–2028. 30418471 10.1001/jama.2018.14854 PMC9582631

[40] Schmitz KH, Courneya KS, Matthews C, Demark-Wahnefried W, Galvao DA, Pinto BM, Irwin ML, Wolin KY, Segal RJ, Lucia A : American College of Sports Medicine roundtable on exercise guidelines for cancer survivors. Med Sci Sports Exerc 2010, 42(7):1409–1426. 20559064 10.1249/MSS.0b013e3181e0c112

[41] Kushi LH, Doyle C, McCullough M, Rock CL, Demark-Wahnefried W, Bandera EV, Gapstur S, Patel AV, Andrews K, Gansler T : American Cancer Society Guidelines on nutrition and physical activity for cancer prevention: reducing the risk of cancer with healthy food choices and physical activity. CA Cancer J Clin 2012, 62(1):30–67. 22237782 10.3322/caac.20140

[42] WHO guidelines on physical activity and sedentary behaviour. Geneva: World Health Organization; 2020. Licence: CC BY-NC-SA 3.0 IGO.

[43] Phipps AI, Shi Q, Zemla TJ, Dotan E, Gill S, Goldberg RM, Hardikar S, Jahagirdar B, Limburg PJ, Newcomb PA : Physical Activity and Outcomes in Patients with Stage III Colon Cancer: A Correlative Analysis of Phase III Trial NCCTG N0147 (Alliance). Cancer epidemiology, biomarkers & prevention : a publication of the American Association for Cancer Research, cosponsored by the American Society of Preventive Oncology 2018, 27(6):696–703. 10.1158/1055-9965.EPI-17-0769 PMC5984710 29563133

[44] Hardikar S, Newcomb PA, Campbell PT, Win AK, Lindor NM, Buchanan DD, Makar KW, Jenkins MA, Potter JD, Phipps AI: Prediagnostic Physical Activity and Colorectal Cancer Survival: Overall and Stratified by Tumor Characteristics. Cancer epidemiology, biomarkers & prevention : a publication of the American Association for Cancer Research, cosponsored by the American Society of Preventive Oncology 2015, 24(7):1130–1137. 10.1158/1055-9965.EPI-15-0039 PMC4491038 25976417

[45] Kuiper JG, Phipps AI, Neuhouser ML, Chlebowski RT, Thomson CA, Irwin ML, Lane DS, Wactawski-Wende J, Hou L, Jackson RD : Recreational physical activity, body mass index, and survival in women with colorectal cancer. Cancer Causes Control 2012, 23(12):1939–1948. 23053793 10.1007/s10552-012-0071-2 PMC3499635

[46] Das S, Forer L, Schonherr S, Sidore C, Locke AE, Kwong A, Vrieze SI, Chew EY, Levy S, McGue M : Next-generation genotype imputation service and methods. Nat Genet 2016, 48(10):1284–1287. 27571263 10.1038/ng.3656 PMC5157836

[47] Morrison J: https://cran.r-project.org/package=BinaryDosage. 2020.

[48] Abecasis GR, Auton A, Brooks LD, DePristo MA, Durbin RM, Handsaker RE, Kang HM, Marth GT, McVean GA: An integrated map of genetic variation from 1,092 human genomes. Nature 2012, 491(7422):56–65. 23128226 10.1038/nature11632 PMC3498066

[49] IntHout J, Ioannidis JP, Borm GF: The Hartung-Knapp-Sidik-Jonkman method for random effects meta-analysis is straightforward and considerably outperforms the standard DerSimonian-Laird method. BMC Med Res Methodol 2014, 14:25. 24548571 10.1186/1471-2288-14-25 PMC4015721

[50] Cochran WG: The combination of estimates from different experiments. 1954, 10:101–129.

[51] Guido Schwarzer JRC, Rücker Gerta.: Meta-Analysis with R. Switzerland: Springer, Cham; 2015.

[52] Dai JY, Logsdon BA, Huang Y, Hsu L, Reiner AP, Prentice RL, Kooperberg C: Simultaneously testing for marginal genetic association and gene-environment interaction. Am J Epidemiol 2012, 176(2):164–173. 22771729 10.1093/aje/kwr521 PMC3499112

[53] Gauderman WJ, Kim A, Conti DV, Morrison J, Thomas DC, Vora H, Lewinger JP: A Unified Model for the Analysis of Gene-Environment Interaction. American journal of epidemiology 2019, 188(4):760–767. 30649161 10.1093/aje/kwy278 PMC6438805

[54] Gao X, Starmer J, Martin ER: A multiple testing correction method for genetic association studies using correlated single nucleotide polymorphisms. Genet Epidemiol 2008, 32(4):361–369. 18271029 10.1002/gepi.20310

[55] Kawaguchi ES, Kim AE, Lewinger JP, Gauderman WJ: Improved two-step testing of genome-wide gene-environment interactions. bioRxiv 2022:2022.2006.2014.496154. 10.1002/gepi.22509 PMC9974838 36571162

[56] Gauderman WJ, Zhang P, Morrison JL, Lewinger JP: Finding novel genes by testing G × E interactions in a genome-wide association study. Genet Epidemiol 2013, 37(6):603–613. 23873611 10.1002/gepi.21748 PMC4348012

[57] Morrison JL, Gauderman WJ (2020). GxEScanR: Run GWAS/GWEIS Scans Using Binary Dosage Files. R package version 2.0.2. https://CRAN.R-project.org/package=GxEScanR.

[58] Kraft P, Yen YC, Stram DO, Morrison J, Gauderman WJ: Exploiting gene-environment interaction to detect genetic associations. Hum Hered 2007, 63(2):111–119. 17283440 10.1159/000099183

[59] Murcray CE, Lewinger JP, Gauderman WJ: Gene-environment interaction in genome-wide association studies. Am J Epidemiol 2009, 169(2):219–226. 19022827 10.1093/aje/kwn353 PMC2732981

[60] Kooperberg C, Leblanc M: Increasing the power of identifying gene x gene interactions in genome-wide association studies. Genetic epidemiology 2008, 32(3):255–263. 18200600 10.1002/gepi.20300 PMC2955421

[61] Ionita-Laza I, McQueen MB, Laird NM, Lange C: Genomewide weighted hypothesis testing in family-based association studies, with an application to a 100K scan. American journal of human genetics 2007, 81(3):607–614. 17701906 10.1086/519748 PMC1950836

[62] Lewinger JP, Kawaguchi ES, Gauderman WJ: A note on p-value multiple-testing adjustment for two-step genome-wide gene-environment interactions scans. medRxiv 2023. 10.1002/gepi.22509 PMC9974838 36571162

[63] de Bakker PI, Ferreira MA, Jia X, Neale BM, Raychaudhuri S, Voight BF: Practical aspects of imputation-driven meta-analysis of genome-wide association studies. Hum Mol Genet 2008, 17(R2):R122–128. 18852200 10.1093/hmg/ddn288 PMC2782358

[64] Devlin B, Roeder K: Genomic control for association studies. Biometrics 1999, 55(4):997–1004. 11315092 10.1111/j.0006-341x.1999.00997.x

[65] Sun M, Bjorge T, Teleka S, Engeland A, Wennberg P, Haggstrom C, Stocks T: Interaction of leisure-time physical activity with body mass index on the risk of obesity-related cancers: A pooled study. Int J Cancer 2022, 151(6):859–868. 35362551 10.1002/ijc.34011 PMC9546504

[66] Pruim RJ, Welch RP, Sanna S, Teslovich TM, Chines PS, Gliedt TP, Boehnke M, Abecasis GR, Willer CJ: LocusZoom: regional visualization of genome-wide association scan results. Bioinformatics 2010, 26(18):2336–2337. 20634204 10.1093/bioinformatics/btq419 PMC2935401

[67] Diez-Obrero V, Dampier CH, Moratalla-Navarro F, Devall M, Plummer SJ, Diez-Villanueva A, Peters U, Bien S, Huyghe JR, Kundaje A : Genetic Effects on Transcriptome Profiles in Colon Epithelium Provide Functional Insights for Genetic Risk Loci. Cell Mol Gastroenterol Hepatol 2021, 12(1):181–197. 33601062 10.1016/j.jcmgh.2021.02.003 PMC8102177

[68] Jordahl KM, Shcherbina A, Kim AE, Su YR, Lin Y, Wang J, Qu C, Albanes D, Arndt V, Baurley JW : Beyond GWAS of Colorectal Cancer: Evidence of Interaction with Alcohol Consumption and Putative Causal Variant for the 10q24.2 Region. Cancer epidemiology, biomarkers & prevention : a publication of the American Association for Cancer Research, cosponsored by the American Society of Preventive Oncology 2022, 31(5):1077–1089. 10.1158/1055-9965.EPI-21-1003 PMC9081195 35438744

[69] Tian Y, Kim AE, Bien SA, Lin Y, Qu C, Harrison T, Carreras-Torres R, Diez-Obrero V, Dimou N, Drew DA : Genome-Wide Interaction Analysis of Genetic Variants with Menopausal Hormone Therapy for Colorectal Cancer Risk. J Natl Cancer Inst 2022. 10.1093/jnci/djac094 PMC9360460 35512400

[70] Cohen AJ, Saiakhova A, Corradin O, Luppino JM, Lovrenert K, Bartels CF, Morrow JJ, Mack SC, Dhillon G, Beard L : Hotspots of aberrant enhancer activity punctuate the colorectal cancer epigenome. Nat Commun 2017, 8:14400. 28169291 10.1038/ncomms14400 PMC5309719

[71] Lee J, Jolanki O, Kim D, Strattan JS, Kundaje A, Nordström K, Shcherbina A: ENCODE-DCC/atac-seq-pipeline: v1.9.1. 2020.

[72] Lee J, Strattan JS, Shcherbina A, Kagda M, Maurizio PL: ENCODE-DCC/chip-seq-pipeline2: v1.6.1. 2020.

[73] Su YR, Di CZ, Hsu L, Genetics, Epidemiology of Colorectal Cancer C: A unified powerful set-based test for sequencing data analysis of GxE interactions. Biostatistics 2017, 18(1):119–131. 27474101 10.1093/biostatistics/kxw034 PMC5255050

[74] Tu L, Yan B, Peng Z: Common genetic variants (rs4779584 and rs10318) at 15q13.3 contributes to colorectal adenoma and colorectal cancer susceptibility: evidence based on 22 studies. Mol Genet Genomics 2015, 290(3):901–912. 25475391 10.1007/s00438-014-0970-x

[75] Yang H, Gao Y, Feng T, Jin TB, Kang LL, Chen C: Meta-analysis of the rs4779584 polymorphism and colorectal cancer risk. PLoS One 2014, 9(2):e89736. 24586997 10.1371/journal.pone.0089736 PMC3933649

[76] Peters U, Hutter CM, Hsu L, Schumacher FR, Conti DV, Carlson CS, Edlund CK, Haile RW, Gallinger S, Zanke BW : Meta-analysis of new genome-wide association studies of colorectal cancer risk. Hum Genet 2012, 131(2):217–234. 21761138 10.1007/s00439-011-1055-0 PMC3257356

[77] Whiffin N, Hosking FJ, Farrington SM, Palles C, Dobbins SE, Zgaga L, Lloyd A, Kinnersley B, Gorman M, Tenesa A : Identification of susceptibility loci for colorectal cancer in a genome-wide meta-analysis. Hum Mol Genet 2014, 23(17):4729–4737. 24737748 10.1093/hmg/ddu177 PMC4133584

[78] Tanskanen T, van den Berg L, Valimaki N, Aavikko M, Ness-Jensen E, Hveem K, Wettergren Y, Bexe Lindskog E, Tonisson N, Metspalu A : Genome-wide association study and meta-analysis in Northern European populations replicate multiple colorectal cancer risk loci. Int J Cancer 2018, 142(3):540–546. 28960316 10.1002/ijc.31076 PMC6383773

[79] Rakshit S, Bhaskar LVKS: An Intergenic Variant rs4779584 Between SCG5 and GREM1 Contributes to the Increased Risk of Colorectal Cancer: A Meta-Analysis. In: Novel therapeutic approaches for gastrointestinal malignancies Diagnostics and Therapeutic Advances in GI Malignancies. edn. Edited by Nagaraju GP, Peela S. Singapore: Springer; 2020: 159–169.

[80] Jaeger E, Webb E, Howarth K, Carvajal-Carmona L, Rowan A, Broderick P, Walther A, Spain S, Pittman A, Kemp Z : Common genetic variants at the CRAC1 (HMPS) locus on chromosome 15q13.3 influence colorectal cancer risk. Nat Genet 2008, 40(1):26–28. 18084292 10.1038/ng.2007.41

[81] Derynck R, Akhurst RJ, Balmain A: TGF-beta signaling in tumor suppression and cancer progression. Nat Genet 2001, 29(2):117–129. 11586292 10.1038/ng1001-117

[82] Stabile H, Mitola S, Moroni E, Belleri M, Nicoli S, Coltrini D, Peri F, Pessi A, Orsatti L, Talamo F : Bone morphogenic protein antagonist Drm/gremlin is a novel proangiogenic factor. Blood 2007, 109(5):1834–1840. 17077323 10.1182/blood-2006-06-032276

[83] Hedjazifar S, Khatib Shahidi R, Hammarstedt A, Bonnet L, Church C, Boucher J, Bluher M, Smith U: The Novel Adipokine Gremlin 1 Antagonizes Insulin Action and Is Increased in Type 2 Diabetes and NAFLD/NASH. Diabetes 2020, 69(3):331–341. 31882566 10.2337/db19-0701

[84] Kobayashi H, Gieniec KA, Wright JA, Wang T, Asai N, Mizutani Y, Lida T, Ando R, Suzuki N, Lannagan TRM : The Balance of Stromal BMP Signaling Mediated by GREM1 and ISLR Drives Colorectal Carcinogenesis. Gastroenterology 2021, 160(4):1224–1239 e1230. 33197448 10.1053/j.gastro.2020.11.011 PMC7617122

[85] Ren J, Smid M, Iaria J, Salvatori DCF, van Dam H, Zhu HJ, Martens JWM, Ten Dijke P: Cancer-associated fibroblast-derived Gremlin 1 promotes breast cancer progression. Breast Cancer Res 2019, 21(1):109. 31533776 10.1186/s13058-019-1194-0 PMC6751614

[86] Corsini M, Moroni E, Ravelli C, Andres G, Grillo E, Ali IH, Brazil DP, Presta M, Mitola S: Cyclic adenosine monophosphate-response element-binding protein mediates the proangiogenic or proinflammatory activity of gremlin. Arterioscler Thromb Vasc Biol 2014, 34(1):136–145. 24233491 10.1161/ATVBAHA.113.302517

[87] Seidah NG, Chretien M: Proprotein and prohormone convertases: a family of subtilases generating diverse bioactive polypeptides. Brain Res 1999, 848(1–2):45–62. 10701998 10.1016/s0006-8993(99)01909-5

[88] Mbikay M, Seidah NG, Chretien M: Neuroendocrine secretory protein 7B2: structure, expression and functions. Biochem J 2001, 357(Pt 2):329–342. 11439082 10.1042/0264-6021:3570329 PMC1221959

[89] Ziai J, Matloff E, Choi J, Kombo N, Materin M, Bale AE: Defining the polyposis/colorectal cancer phenotype associated with the Ashkenazi GREM1 duplication: counselling and management recommendations. Genet Res (Camb) 2016, 98:e5. 26947005 10.1017/S0016672316000021 PMC6865171

[90] Yusuf I, Pardamean B, Baurley JW, Budiarto A, Miskad UA, Lusikooy RE, Arsyad A, Irwan A, Mathew G, Suriapranata I : Genetic risk factors for colorectal cancer in multiethnic Indonesians. Sci Rep 2021, 11(1):9988. 33976257 10.1038/s41598-021-88805-4 PMC8113452

[91] Jo Y, Yeo MK, Dao T, Kwon J, Yi HS, Ryu D: Machine learning-featured Secretogranin V is a circulating diagnostic biomarker for pancreatic adenocarcinomas associated with adipopenia. Front Oncol 2022, 12:942774. 36059698 10.3389/fonc.2022.942774 PMC9428794

[92] Farber CR, Chitwood J, Lee SN, Verdugo RA, Islas-Trejo A, Rincon G, Lindberg I, Medrano JF: Overexpression of Scg5 increases enzymatic activity of PCSK2 and is inversely correlated with body weight in congenic mice. BMC Genet 2008, 9:34. 18439298 10.1186/1471-2156-9-34 PMC2386500

[93] Klimentidis YC, Raichlen DA, Bea J, Garcia DO, Wineinger NE, Mandarino LJ, Alexander GE, Chen Z, Going SB: Genome-wide association study of habitual physical activity in over 377,000 UK Biobank participants identifies multiple variants including CADM2 and APOE. Int J Obes (Lond) 2018, 42(6):1161–1176. 29899525 10.1038/s41366-018-0120-3 PMC6195860

[94] Doherty A, Smith-Byrne K, Ferreira T, Holmes MV, Holmes C, Pulit SL, Lindgren CM: GWAS identifies 14 loci for device-measured physical activity and sleep duration. Nat Commun 2018, 9(1):5257. 30531941 10.1038/s41467-018-07743-4 PMC6288145

[95] Fabre O, Giordani L, Parisi A, Pattamaprapanont P, Ahwazi D, Brun C, Chakroun I, Taleb A, Blais A, Andersen E : GREM1 is epigenetically reprogrammed in muscle cells after exercise training and controls myogenesis and metabolism. bioRxiv 2020:2020.2002.2020.956300.

[96] Jurdana M: Physical activity and cancer risk. Actual knowledge and possible biological mechanisms. Radiol Oncol 2021, 55(1):7–17. 33885236 10.2478/raon-2020-0063 PMC7877262

[97] Wang T, Zhang Y, Taaffe DR, Kim JS, Luo H, Yang L, Fairman CM, Qiao Y, Newton RU, Galvao DA: Protective effects of physical activity in colon cancer and underlying mechanisms: A review of epidemiological and biological evidence. Crit Rev Oncol Hematol 2022, 170:103578. 35007701 10.1016/j.critrevonc.2022.103578

[98] Dziewiecka H, Buttar HS, Kasperska A, Ostapiuk-Karolczuk J, Domagalska M, Cichon J, Skarpanska-Stejnborn A: Physical activity induced alterations of gut microbiota in humans: a systematic review. BMC Sports Sci Med Rehabil 2022, 14(1):122. 35799284 10.1186/s13102-022-00513-2 PMC9264679

[99] Saeidi A, Seifi-Ski-Shahr F, Soltani M, Daraei A, Shirvani H, Laher I, Hackney AC, Johnson KE, Basati G, Zouhal H: Resistance training, gremlin 1 and macrophage migration inhibitory factor in obese men: a randomised trial. Arch Physiol Biochem 2020:1–9. 10.1080/13813455.2020.1856142 33370549

[100] Ataeinosrat A, Saeidi A, Abednatanzi H, Rahmani H, Daloii AA, Pashaei Z, Hojati V, Basati G, Mossayebi A, Laher I : Intensity Dependent Effects of Interval Resistance Training on Myokines and Cardiovascular Risk Factors in Males With Obesity. Front Endocrinol (Lausanne) 2022, 13:895512. 35757424 10.3389/fendo.2022.895512 PMC9226680

[101] Pourteymour S, Eckardt K, Holen T, Langleite T, Lee S, Jensen J, Birkeland KI, Drevon CA, Hjorth M: Global mRNA sequencing of human skeletal muscle: Search for novel exercise-regulated myokines. Mol Metab 2017, 6(4):352–365. 28377874 10.1016/j.molmet.2017.01.007 PMC5369209

[102] Saran U, Guarino M, Rodriguez S, Simillion C, Montani M, Foti M, Humar B, St-Pierre MV, Dufour JF: Anti-tumoral effects of exercise on hepatocellular carcinoma growth. Hepatol Commun 2018, 2(5):607–620. 29761175 10.1002/hep4.1159 PMC5944574

[103] Endo Y, Zhang Y, Olumi S, Karvar M, Argawal S, Neppl RL, Sinha I: Exercise-induced gene expression changes in skeletal muscle of old mice. Genomics 2021, 113(5):2965–2976. 34214629 10.1016/j.ygeno.2021.06.035 PMC8403630

[104] Entrez Gene: KCNG1 potassium voltage-gated channel, subfamily G, member 1. Available at https://www.ncbi.nlm.nih.gov/gene/3755. In.

[105] Bray MS, Hagberg JM, Perusse L, Rankinen T, Roth SM, Wolfarth B, Bouchard C: The human gene map for performance and health-related fitness phenotypes: the 2006–2007 update. Med Sci Sports Exerc 2009, 41(1):35–73. 19123262 10.1249/mss.0b013e3181844179

[106] Wong HL, Koh WP, Probst-Hensch NM, Van den Berg D, Yu MC, Ingles SA: Insulin-like growth factor-1 promoter polymorphisms and colorectal cancer: a functional genomics approach. Gut 2008, 57(8):1090–1096. 18308828 10.1136/gut.2007.140855 PMC2752962

[107] Ke J, Lou J, Chen X, Li J, Liu C, Gong Y, Yang Y, Zhu Y, Zhang Y, Gong J: Identification of a Potential Regulatory Variant for Colorectal Cancer Risk Mapping to Chromosome 5q31.1: A Post-GWAS Study. PLoS One 2015, 10(9):e0138478. 26381143 10.1371/journal.pone.0138478 PMC4575091

[108] Liu DX, Lobie PE: Transcriptional activation of p53 by Pitx1. Cell Death Differ 2007, 14(11):1893–1907. 17762884 10.1038/sj.cdd.4402209

[109] Prince SA, Adamo KB, Hamel ME, Hardt J, Connor Gorber S, Tremblay M: A comparison of direct versus self-report measures for assessing physical activity in adults: a systematic review. Int J Behav Nutr Phys Act 2008, 5:56. 18990237 10.1186/1479-5868-5-56 PMC2588639

